# IQ-TREE 2: New Models and Efficient Methods for Phylogenetic Inference in the Genomic Era

**DOI:** 10.1093/molbev/msaa015

**Published:** 2020-02-03

**Authors:** Bui Quang Minh, Heiko A Schmidt, Olga Chernomor, Dominik Schrempf, Michael D Woodhams, Arndt von Haeseler, Robert Lanfear

**Affiliations:** m1 Research School of Computer Science, Australian National University, Canberra, ACT, Australia; m2 Department of Ecology and Evolution, Research School of Biology, Australian National University, Canberra, ACT, Australia; m3 Center for Integrative Bioinformatics Vienna, Max Perutz Labs, University of Vienna and Medical University of Vienna, Vienna, Austria; m4 Department of Biological Physics, Eötvös Lórand University, Budapest, Hungary; m5 Discipline of Mathematics, University of Tasmania, Hobart, TAS, Australia; m6 Bioinformatics and Computational Biology, Faculty of Computer Science, University of Vienna, Vienna, Austria

**Keywords:** phylogenetics, phylogenomics, maximum likelihood, models of sequence evolution

## Abstract

IQ-TREE (http://www.iqtree.org, last accessed February 6, 2020) is a user-friendly and widely used software package for phylogenetic inference using maximum likelihood. Since the release of version 1 in 2014, we have continuously expanded IQ-TREE to integrate a plethora of new models of sequence evolution and efficient computational approaches of phylogenetic inference to deal with genomic data. Here, we describe notable features of IQ-TREE version 2 and highlight the key advantages over other software.

IQ-TREE is a widely used and open-source software package for phylogenetic inference using the maximum likelihood (ML) criterion. The high performance of IQ-TREE results from the efficient integration of novel phylogenetic methods that improve the three key steps in phylogenetic analysis: fast model selection via ModelFinder (Kalyaanamoorthy et al. 2017), an effective tree search algorithm (Nguyen et al. 2015), and a novel ultrafast bootstrap approximation (Minh et al. 2013; Hoang et al. 2018). Zhou et al. (2018) independently showed that the tree search algorithm in IQ-TREE exhibits good performance in terms of both computing times and likelihood maximization when compared with other popular ML phylogenetics software such as RAxML (Stamatakis 2014) and PhyML (Guindon et al. 2010). IQ-TREE also plays a vital role in the software ecosystem for biomedical research. For instance, it is an integral component of many popular open-source applications such as Galaxy (Afgan et al. 2018), Nextstrain (Hadfield et al. 2018), OrthoFinder (Emms and Kelly 2015), and QIIME 2 (Bolyen et al. 2019).

Since the release of IQ-TREE version 1.0 in 2014, we have continuously developed IQ-TREE to integrate a plethora of new evolutionary models and efficient methods for analyzing large phylogenomic data sets. Here, we present IQ-TREE version 2 and highlight the key new features and improvements. To demonstrate its performance and compare it with other software, we used a 2-GHz CPU server to analyze 2 large sequence alignments: a DNA alignment of 110 vertebrate species and 25,919 sites (Fong et al. 2012) which we call the DNA-data set, and an amino acid alignment of 76 metazoan species and 49,388 sites (Whelan et al. 2017) which we call the AA-data set.

## Time-Reversible Models of Sequence Evolution

IQ-TREE 2 supports more than 200 time-reversible evolutionary models, including all standard substitution models for DNA, protein, codon, binary, and multistate-morphological data (Felsenstein 2004; Lemey et al. 2009). Rate heterogeneity across sites can be accommodated either by the discrete Γ distribution (Yang 1994b) with invariant sites (Gu et al. 1995) or by a distribution-free rate model (Kalyaanamoorthy et al. 2017). Site-specific rates can be estimated by the empirical Bayesian method via the --rate option or by ML (Mayrose et al. 2004) via the --mlrate option. These estimated site-specific rates can be useful for downstream analysis such as quantification of phylogenetic informativeness, signal, and noise (Dornburg et al. 2016). For single nucleotide polymorphism or morphological data, the absence of invariant sites can be accounted for by an ascertainment bias correction (Lewis 2001).

Moreover, IQ-TREE 2 offers a number of advanced models for phylogenomic data including partitioned models (Lanfear et al. 2012; Chernomor et al. 2016), mixture models (Le et al. 2008, 2012; Le and Gascuel 2010), posterior-mean site frequency models (Wang et al. 2018), and heterotachy models (Crotty et al. 2019). For allele-frequency data, IQ-TREE 2 implements polymorphism-aware models (Schrempf et al. 2016, 2019). With partitioned models, one can specify either a partition file (as in IQ-TREE 1) or a directory of single-locus alignments (a new feature in IQ-TREE 2). In the latter case, IQ-TREE 2 will load and concatenate all alignments within the directory, eliminating the need for users to manually perform this step. In addition to implementing existing mixture models, IQ-TREE 2 goes beyond the mixture models employed in PhyML-mixtures (Le et al. 2008) and RAxML-NG software (Kozlov et al. 2019), by allowing for user-defined mixture models using the “MIX{model_1_,…,model_k_}” syntax. IQ-TREE 2 is also substantially faster than PhyML-mixtures. For example, optimization of the model parameters for the LG4X model on the AA-data set took 1.9 min in IQ-TREE 2, 3 min in RAxML-NG, and 17.6 min in PhyML-mixtures.

Because the aforementioned substitution models assume time reversibility, IQ-TREE 1 only enabled inference of *unrooted* trees (Felsenstein 1981). In IQ-TREE 2, we included non-time-reversible models (e.g., Norris 1997), meaning that IQ-TREE 2 enables inference of *rooted* trees.

## Nonreversible Substitution Models

IQ-TREE 2 allows users to reconstruct rooted trees using nonreversible models, a feature not available in most ML packages due to numerical and computational expense. We substantially revised the IQ-TREE code to overcome these obstacles. First, due to nonreversibility of the rate matrix Q, the naïve computation of the transition probability matrix $P\left(t\right)=e^{Qt}$, where t is the branch length, is unstable due to complex eigenvalues of Q (Moler and Loan 1978). Eigen-decomposition and scaling-squaring techniques, for example, as provided in the Eigen3 library (Guennebaud et al. 2010) remove the numerical problems. IQ-TREE 2 employs eigen-decomposition to diagonalize Q into its (complex) eigenvalues, eigenvectors and inverse eigenvectors, which are used to compute $P\left(t\right)$. If Q is not diagonalizable, then IQ-TREE 2 switches to the scaling-squaring technique to compute $P\left(t\right)$. The eigen-decomposition is fast but sometimes unstable, whereas the scaling-squaring is slow but stable. Second, IQ-TREE 2 uses a rooted tree data structure and an adjusted pruning algorithm for computing likelihoods of nonreversible models on rooted trees (Boussau and Gouy 2006). Third, we adapted the hill climbing nearest neighbor interchange, part of the tree search heuristic in IQ-TREE to account for rooted trees. Fourth, we introduced a root search operation that moves the root to the neighboring branches (by default up to 2 branches away from the current root branch) and retains the rooting position with the highest likelihood. Users can increase this parameter (--root-dist option) to test for the position of the root across more or all branches.

Based on these improvements, we could efficiently implement 99 nonreversible DNA models known as Lie Markov models (Woodhams et al. 2015), the unrestricted model (UNREST) for DNA (Yang 1994a) and—for the first time—the general nonreversible model for amino acid sequences that we call NONREV. For the DNA-data set (Fong et al. 2012), tree search under the general time-reversible model took 2.8 min using 4 CPU cores, whereas the UNREST model took 10.7 min. For the AA-data set (Whelan et al. 2017) tree search under the protein general time-reversible model took 2.52 h, whereas the NONREV model took 5.8 h. The implementation of nonreversible models in IQ-TREE 2 opens new avenues of evolutionary research.

## Fast Likelihood Mapping Analysis

IQ-TREE 2 provides a fast and parallel implementation of the quartet likelihood mapping (Strimmer and von Haeseler 1997) to visualize phylogenetic information in alignments or to study the relationships of taxon-groups in large data sets. To this end, IQ-TREE 2 evaluates the exact ML value of all relevant quartets. Application of the original implementation of quartet likelihood mapping in TREE-PUZZLE (Schmidt et al. 2002) (i.e., with 10,000 random quartets and exact ML quartet evaluation) to the DNA-data set took 282 min, whereas the improved implementation in IQ-TREE 2 required only 1 min using one CPU core and 21 s using four cores. Similarly, analysis of the AA-data set took 99.5 h in TREE-PUZZLE, 17 min in IQ-TREE 2 with one CPU core, and 4.5 min using four cores. Together with the extended repertoire of sequence evolution models, likelihood mapping facilitates a thorough investigation of much larger sequence alignments.

## New Options for Tree Search

IQ-TREE 2 allows users to perform a constrained tree search (-g option), such that the resulting ML tree will respect a set of user-defined splits, which may also contain polytomies. This option is helpful to enforce the monophyly of certain groups. A tree test (see below) can be performed to ensure that the constrained tree is not significantly worse than the unconstrained tree.

IQ-TREE 2 also provides a fast tree search (-fast option) using an algorithm resembling that implemented in FastTree2 (Price et al. 2010). Here, IQ-TREE 2 computes two starting trees using Maximum Parsimony and Neighbor-Joining (Gascuel 1997), which are then optimized by the hill climbing nearest neighbor interchange moves. For our example DNA-data set, the default tree search took 27 min, whereas the fast IQ-TREE search and FastTree2 needed 82 and 85 s, respectively. For the AA-data set, the default tree search took 5.7 h, whereas the fast IQ-TREE search and the FastTree2 search took 13.9 and 4.3 min, respectively. The speed of FastTree2 was accomplished at the cost of producing substantially worse trees than RAxML, PhyML, and IQ-TREE (Zhou et al. 2018).

IQ-TREE 2 also provides a --runs option, which conducts multiple independent tree searches, summarizes the resulting trees, and reports the tree(s) with the highest log-likelihood. This option is recommended for difficult data sets, for example, with many taxa and/or limited phylogenetic signal, to increase the probability that the true ML tree is found.

## Systematically Accounting for Missing Data

Missing data—when some loci or sites are absent for some species—are almost unavoidable in phylogenomic data sets. In the presence of missing data, each species tree can be associated with a corresponding set of induced single-locus trees. For each locus, an induced tree is obtained from the species tree by removing species with unavailable sequences for that locus. Missing data can create phylogenetic terraces (Sanderson et al. 2011), where for two or more species trees the associated sets of induced single-locus trees are identical, leading to identical likelihoods under an edge-unlinked partitioned model.

IQ-TREE 2 employs the terraphast library (Biczok et al. 2018) to automatically report the inferred ML trees that reside on a terrace. If so, users are advised to gather more data or filter out gappy taxa/loci. Moreover, IQ-TREE 2 generalizes the terrace concept to partial terraces (Chernomor et al. 2015), where a subset of the induced single-locus trees is identical. IQ-TREE 2 exploits partial terraces to improve tree search under partitioned models and achieves up to 4.5- and 8-fold speedups compared with IQ-TREE 1 and RAxML, respectively (Chernomor et al. 2016).

## Single-Locus Tree Inference

IQ-TREE 2 enables users to infer individual locus trees (-S option), which can be used for subsequent coalescent (e.g., Mirarab et al. 2014) or concordance analyses (e.g., Minh et al. 2018). Users need to specify either a partition file that delineates the borders between loci or a directory containing the individual locus alignments in individual files. In both cases, IQ-TREE 2 will perform separate model selection and tree searches for each locus, which are automatically scheduled on the *k* CPU cores. The loci are ranked according to their expected computational costs, where the costs are estimated as a product of the number of sequences, distinct site patterns, and character states (4 for DNA and 20 for protein). The *k* loci with highest costs will be assigned to one of the *k* cores. When a core has finished computation the next locus in the ranked list will be assigned to that core. This process continues until all computations are complete.

We compared our scheduling approach with the program ParGenes version 1.0.1 (Morel et al. 2019), which uses RAxML-NG for tree search and a more sophisticated scheduling algorithm. For the DNA-data set, IQ-TREE 2 inferred 168 locus trees in 18.6 and 9.2 min using 2 and 4 CPU cores, respectively; whereas the same analysis in ParGenes took 9.17 and 5.98 min. For the AA-data set with 127 loci, IQ-TREE 2 took 2.05 and 1.05 h using 2 and 4 cores, respectively; whereas ParGenes needed 1.53 and 0.81 h. Therefore, IQ-TREE 2 shows a higher parallel efficiency (DNA: 101% and AA: 97.6%) (Grama et al. 2003) than ParGenes (DNA: 76.7% and AA: 94.4%), but ParGenes needs less computational time. The upshot of this is that both programs are likely to perform similarly on very large data sets.

## Fast Branch Tests

IQ-TREE 2 provides fast and parallel implementations for several existing branch tests including the approximate likelihood ratio test (aLRT) (Anisimova and Gascuel 2006), the Shimodaira–Hasegawa-like aLRT (SH-aLRT) (Guindon et al. 2010), and the aBayes test (Anisimova et al. 2011). The SH-aLRT is parallelized over the bootstrap samples to maximize load balance and efficiency. These tests can be performed on the reconstructed ML tree or a user-defined tree. For the DNA-data set, PhyML version 3.3.20190321 (Guindon et al. 2010) took 33.9 h to perform the SH-aLRT, whereas IQ-TREE 2 needed only 1.5 min (1,300-fold speedup). On the AA-data set, PhyML took 173.5 h to perform the SH-aLRT tests, whereas IQ-TREE 2 needed just 1.6 min (a greater than 2,000-fold speedup).

## Fast Topology Tests

IQ-TREE 2 also provides fast and parallel implementations of existing tree topology tests including the Shimodaira–Hasegawa test (Shimodaira and Hasegawa 1999), the approximately unbiased test (Shimodaira 2002), and the expected likelihood weight (Strimmer and Rambaut 2002). Moreover, IQ-TREE 2 is well suited for partitioned models because it provides the site, gene, and gene-site bootstrap resampling schemes (Hoang et al. 2018).

For the DNA-data set CONSEL (Shimodaira and Hasegawa 2001), the original and unparallelized implementation of the approximately unbiased test, took 5 min to test 100 tree topologies, whereas IQ-TREE 2 took 1.8 min with one CPU core and 1.1 min with four CPU cores. For the AA-data set, CONSEL took 9.4 min and IQ-TREE 2 took 8.7 min with one CPU core and 2.4 min with four CPU cores. IQ-TREE provides added convenience by calculating site log-likelihoods itself, rather than relying on site log-likelihood output provided by other software.

## Scalability with Large Data Sets

IQ-TREE 2 implements several features to facilitate the analysis of large data sets. It uses multithreading to speed up computations in a range of areas and can automatically determine the best number of threads for the computer at hand. IQ-TREE 2 also parallelizes the computation across cluster nodes using Message Passing Interface (Snir et al. 1998). IQ-TREE 2 periodically writes a compressed checkpoint file that enables resumption of an interrupted analysis. IQ-TREE 2 also provides a memory-saving mode (Izquierdo-Carrasco et al. 2012), that is automatically invoked when the memory requirement exceeds the RAM size, and a safe mode (-safe option) to avoid numerical underflow for taxon-rich alignments (automatically invoked for data sets with >2,000 sequences).

We benchmarked the memory-saving mode, where the RAM consumption is reduced by half (-mem 0.5). For the DNA-data set, IQ-TREE 2 took 27 and 32.8 m (21% increase) under the full- and half-memory mode, respectively, whereas the same analysis needed 5.7 and 6.2 h (9% increase) for the AA-data set. This increase in computing times is insignificant compared with 50% saving in memory, which could otherwise be a bottleneck for very large data sets and mixture models requiring hundreds of GB of RAM.

## Documentation, User Support, and Workshop Materials

An extensive user manual, quick start guide, tutorials, and command reference are available (http://www.iqtree.org/doc, last accessed February 6, 2020). We actively maintain a forum (https://groups.google.com/d/forum/iqtree, last accessed February 6, 2020) for user support, bug reports, and feature requests. We regularly teach IQ-TREE at the Workshop on Molecular Evolution (https://molevolworkshop.github.io, last accessed February 6, 2020), the Workshop on Virus Evolution and Molecular Epidemiology (https://rega.kuleuven.be/cev/veme-workshop/, last accessed February 6, 2020), and the Workshop on Phylogenomics (http://evomics.org, last accessed February 6, 2020). Most workshop materials are freely available at http://www.iqtree.org/workshop/ (last accessed February 6, 2020).

## Supplementary Material

Supplementary data are available at *Molecular Biology and Evolution* online.

## Supplementary Material

msaa015_Supplementary_Data
