# Corrigendum to: IQ-TREE 2: New Models and Efficient Methods for Phylogenetic Inference in the Genomic Era

**DOI:** 10.1093/molbev/msaa131

**Published:** 2020-06-18

**Authors:** Bui Quang Minh, Heiko A Schmidt, Olga Chernomor, Dominik Schrempf, Michael D Woodhams, Arndt von Haeseler, Robert Lanfear

*Mol. Biol. Evol*. doi:10.1093/molbev/msaa015

The acknowledgments section of this paper has been expanded since original publication to include the European Research Council under the European Union’s Horizon 2020 research and innovation programme (Grant No. 714774) to D.S.

