# Supplementary material for: IQ-TREE 2: New Models and Efficient Methods for Phylogenetic Inference in the Genomic Era
Source: Mol Biol Evol. 2020 Feb 3;37(5):1530–4. doi: 10.1093/molbev/msaa015 (PMC7182206; doi:10.1093/molbev/msaa015)
Supplement: msaa015_Supplementary_Data [file msaa015_supplementary_data.zip › msaa015-Suppl_Data/IQTree2-response.pdf]

Editors' comments to the author:

The comments and recommendations from two expert reviewers are now available for your manuscript. These reviewers judged the reported discoveries tools to be of high significance, and the potential community impact of your work to be high. They found that the manuscript needs some improvement in text and a small amount of benchmarking. Editors generally agree with their concerns and recommendations, which led to a designation of high priority.

The recommendation is given assuming that you will be able to satisfactorily address all the reviewers' comments. Without a satisfactory response, the priority may decline in the next round of review/consideration.

Answer: We thank Editor and Reviewers for the positive judgement on the manuscript. We have now addressed all the comments and provide the point-to-point responses highlighted in red below. We also labelled corresponding changes in the revision, so that the Reviewer can cross reference them quickly. Moreover, we added a benchmark for the memory-saving feature in the section "Scalability with large datasets", as this is critical for very large datasets. We hope that the manuscript is now suitable for publication in Molecular Biology and Evolution.

Reviewers' comments:

Reviewer: 1

Comments to the Author
The authors present a summary of the novel features implemented in IQ-Tree which by now has become a widely used tool for phylogenetic inference under the Maximum Likelihood model. As the last publication on IQ-Tree was published 5 years ago, the authors have chosen a good point in time to publish an update. Given, the immense effort that was invested into maintaining, improving, and extending the software (in particular by numerically challenging and otherwise tricky non-reversible models), the article should be published.

I only have two minor comments/remarks for a revision:

1. IQ-Tree version 1.6 did not receive such a good code quality score in the code quality benchmark tool we have recently developed
(see https://github.com/adrianzap/softwipe/wiki/Code-Quality-Benchmark). I know that the authors are aware of this and I would appreciate if they could calculate the code quality rank for IQ-Tree version 2 (we are willing to help with this) and mention it in the paper, as I believe that this is an important issue, especially for such a widely used tool.

Answer: We requested Alexandros Stamatakis' team to perform the code quality analysis and update that website. IQ-TREE 1.6.10 received a score of 5.2, whereas IQ-TREE 2 has a slightly higher score of 6. We added a reference to this website in the Acknowledgement without the detailed scores, because this tool and the benchmark results were not published and thus cannot be citable elsewhere in the manuscript.

2. In paragraph "Single Locus Tree Inference" I believe that the authors should mention our recently published and very similar tool for that purpose called
ParGenes https://academic.oup.com/bioinformatics/article/35/10/1771/5132696
In particular, they should discuss in a bit more depth, the scheduling algorithm they use and mention how it performs compared to ParGenes, as the scheduling and optimal resource allocation and utilization constitute the tricky parts of this specific tree inference problem.

I hope this helps.

Answer: We added a description of the scheduling algorithm in IQ-TREE 2 and a benchmark with ParGenes.

Reviewer: 2

Comments to the Author
This manuscript presents a revised and empowered version of IQ-TREE, IQ-TREE 2, currently one of the most popular (and fast) packages for performing maximum likelihood inference of phylogenetic trees. The new version includes implementations of a large number of new sequence evolutionary models with expanded applicability to large phylogenomic datasets, while maintaining or improving its marked advantage with computational speed. New features include time-reversible models of sequence evolution, construction of rooted trees using non-reversible models, extra fast quick search options, fast and parallel quartet likelihood mapping, constrained tree search and splits testing, and increased scalability. Extensive documentation, user support, and workshop materials are provided. These represent a significant advance and are well worthy of publication.

I have a number of minor suggestions (mostly suggested edits to the writing, some of which are fairly stylistic and should be optional to the authors) meant to improve the clarity and impact of the manuscript:

Answer: We thank you for the thorough reading of the manuscript. All suggestions for rephrasing have been incorporated. In the following we only provide answers to the other comments.

P3
L53 IQ-TREE 2 ^accommodates / supports^ more than…
L56 models^, including^ all standard substitution models

P4
L4 It is not mentioned, but of significant interest, that IQ-TREE 2 enables distribution-free estimation of site rates, a feature that can be used for quantification of phylogenetic informativeness, signal, and noise. This advance should be reported. Does IQ-TREE 2 use the Kalyaanamoorthy (2017) probability-distribution free model to do this calculation, or calculate rates directly using maximum likelihood on the site states and a fixed tree?

Answer: We added to the manuscript the following sentences: Site-specific rates can be estimated by the empirical Bayesian method via the --rate option or by maximum likelihood (Mayrose et al. 2004) via the --mlrate option. These estimated site-specific rates can useful for downstream analysis such as quantification of phylogenetic informativeness, signal and noise (Dornburg et al. 2016).

L6–7 please explain with more detail and clarity.

Answer: An explanation would require extended text and thus exceeds the word limits. Therefore, we think that a reference (Lewis 2001) is enough.

L11 clarify membership in “200 models” mentioned above

Answer: These models are not included in the 200 models above. We added “Moreover,…” to make that point.

L18 For ^allele-frequency data, IQ-TREE implements polymorphism-aware^ models
L25 latter case^,^ IQ-TREE 2

L27 step.^In addition to implementing the mixture model, the implementation in IQ-TREE goes beyond mixtures employing in PhyML^
L32 RAxML-NG ^(Kozlov et al. 2019), IQ-TREE 2 provides^ user-defined
L32 How are the user-defined mixture models defined?

Answer: We added the syntax to specify user-defined models.

L34 PhyML-mixtures^. For example,^ optimisation
L41 does the term “substitution model” necessarily presume time-reversibility??

Answer: No. We changed it to “aforementioned substitution models”, that explicitly refer to the previously described models.

L41 only ^enabled inference of^ unrooted trees
L44 IQ-TREE 2^,^ we
L44 be more specific about causation / model classification here

Answer: We additionally cited a book (Norris 1997) that clarified non-time-reversible models.

L46 TREE 2 ^enables inference of^ rooted trees.

Answer: Lines 41-46 have now been edited accordingly.

P5
L43 and^—for the first time—^the
L36 ^,^ tree

Answer: L36 is a typo and it actually refers to L46.

L48 define the specific hardware being used for benchmarking once before referring to benchmark times. Also, use SI units “s”, “min”, “h” in reporting benchmarking times throughout.

Answer: We additionally described the hardware in the Introduction (page 3) and adopted SI units throughout the text.

P6
L4 of ^quartet likelihood^ mapping
L10 quartets. ^Application of^ the original
L13 of ^quartet^ likelihood
L15 evaluation) ^to the DNA-dataset^ took
L37–41 groups. ^A tree test (see below) can be performed^ to ensure
L46 option) ^using an algorithm resembling that implemented in FastTree2^ (Price et al. 2010). IQ-TREE 2 computes^ two starting trees
L60 respectively. ^The speed of^

P7
L3 ^FastTree2 is accomplished at the cost of producing^ substantially worse trees
L6–7 “this fast option”: ambiguously written, what does it refer to? Maybe just delete this sentence.

Answer: We deleted this sentence as suggested.

L23 data^—^when … species^—^are
L28 trees induce trees? I know what is meant here but find this shorthand more confusing than illuminating. Take the time to write out a clear explanation.

Answer: We clarified this point by rephrasing and adding two more sentences for the explanation.

L33 automatically ^report the inferred ML trees that reside^ on a terrace
L35 If ^so, users are advised to gather more data or filter^ out gappy taxa/loci.
L40–42 “where … trees”: tree have trees? shorthand language again too compressed to be illuminating. Write it out clearly.

Answer: Rephrased.

L52 IQ-TREE 2 ^enables^ users
L57 2018). ^^ Users

P9
L6 IQ-TREE ^provides added convenience by calculating” site log-likelihoods
L23 that ^enables resumption of^ an interrupted analysis.
L25 also ^provides a memory-saving^ mode
